# Profile of tuberculosis among the foreign-born population in Japan, 2007–2014

**DOI:** 10.5365/WPSAR.2016.7.1.008

**Published:** 2016-06-15

**Authors:** Lisa Kawatsu, Kazuhiro Uchimura, Kiyohiko Izumi, Akihiro Ohkado, Nobukatsu Ishikawa

**Affiliations:** a The Research Institute of Tuberculosis, Japan, Anti-Tuberculosis Association, Tokyo, Japan.; b Graduate School of Biomedical Sciences, Nagasaki University, Nagasaki, Japan.

## Abstract

The proportion of foreign-born people among the newly notified tuberculosis (TB) patients has been increasing in recent years and potentially poses a new challenge to TB control in Japan. In this report, we analysed the data from the Japan TB surveillance system between 2007 and 2014 to gain an overview of the trends and characteristics of foreign-born TB patients in Japan.

We found that the proportion of foreign-born TB patients was especially high among the younger age groups – 44.1% among the 20–29 years age group in 2014. The largest groups of foreign-born patients were from China and the Philippines; however, the number of those from Nepal and Viet Nam was on the rise. Students comprised the second largest professional category group for TB after regular workers, and its proportion increased over the study period. Compared to Japan-born TB patients, foreign-born patients were more likely to be diagnosed through routine medical check-ups. Treatment successes and patients still on treatment were significantly lower among foreign-born patients than their Japan-born counterparts; and transferred-out and unknown outcomes were higher. Our results indicated that distinctive subgroups within the foreign-born population in Japan, especially students and regular workers, might have a higher risk of developing TB. Measures to ensure early diagnosis and treatment adherence should be adapted to such populations.

## Introduction

In many tuberculosis (TB) low-incidence countries, foreign-born people bear a disproportionate burden of the disease. Studies have indicated that these people often encounter various socioeconomic, cultural and behavioural challenges in their host countries that not only increase their risk of developing TB but also delay diagnosis and poor treatment outcomes. (*1*, *2*) In Japan, a TB middle-burden country, the notification rate was 15.4 per 100 000 population in 2014 with foreign-born TB patients contributing 5.8% to the total newly notified cases. (*3*) The proportion has been increasing and potentially poses a new challenge to TB control in Japan. (*4*)

Japan introduced its first nationwide computerized TB surveillance system, the Japan Tuberculosis Surveillance (JTBS), in 1987. TB is a notifiable disease and local public health centres (PHCs) are responsible for entering the data of notified patients into the system. The data are updated every month. Major findings are published annually and are available online. (*5*) Data quality is ensured via the system’s automatic verification programme as well as regular meetings at local levels attended by staff from hospitals and PHCs. Periodic refresher trainings on data entry are also provided to PHC nurses across the nation.

Sound policy-making should be informed by scientific evidence, and a detailed analysis of surveillance data can provide one such resource. In this report, we analysed the data from the JTBS between 2007 and 2014 to gain an overview of the burden of foreign-born TB patients in Japan.

## Methods

We conducted a cross-sectional study whereby aggregated data of newly registered TB cases in the JTBS between 1 January 2007 and 31 December 2014 were analysed. The years 2007 to 2014 were chosen as the study period as the information regarding nationality (either “Japanese” or “non-Japanese”) was added to JTBS in 1998, and country name and year of entry (either “within five years,” or “more than five years” or “unknown”) were only added in 2007. In 2012, the category of nationality was changed to country of birth (either “Japan-born,” “foreign-born” or “unknown”). Definitions of variables are described in detail in **Table 1**. Characteristics of foreign-born TB patients were summarized by number and proportion by sex and age groups; country of birth; professional status; mode of detection; treatment outcome; multidrug resistance (MDR); and status of HIV co-infection. Where appropriate, these characteristics were compared with those of the Japan-born patients. Data of those whose country of birth was “unknown” were excluded from the analysis.

**Table 1:** Definition of variables in the JTBS

Professional category	
Service industry workers	Those involved in interacting with and directly serving customers.
Health-care workers	Doctors, nurses, public health nurses and other medical para-professionals.
Teachers	Teachers of pre-school, junior and high schools and higher education.
Students	Students of junior and high schools and higher education and of private schools, e.g. Japanese language schools.
Regular workers	Those employed full-time on a mid- to long-term contract.
Irregular workers	Those employed part-time, or on short-term contract.
Self-employed	Those self-employed.
Household worker	Those mainly doing housework, e.g. housewives.
Unemployed and others	Those unemployed, including retirees, and those with work other than above.

Mode of detection	
Individual medical check-up	Medical check-up conducted on an individual basis.
Routine medical check-up	Routine medical check-up at schools, workplaces and other social institutions which are mandatory under Japanese law.
Contact investigation	Medical check-up conducted as part of contact investigation.
Other mass medical check-up	Mass medical check-up not mandatory under the law but organized and conducted by local public health centres for specific population considered to be at high risk e.g. students of Japanese language schools, homeless people.
Visit to medical institution/while being hospitalized for other illness	Medical check-up conducted (1) as a result of patient visiting a medical institution (because of any symptoms) or (2) while patient is hospitalized for other illness.
Medical check-up during TB follow-up	Medical check-up conducted as part of follow-up for patients with latent TB infection.

χ^2^ test was conducted to compare proportions for the modes of detection and treatment outcomes among Japan- and foreign-born patients. Age-adjusted rate for treatment outcomes of the two groups were calculated using the 2010 population census data. (*6*) Data trend was evaluated by the Cochran-Armitage test for trend. A *P*-value < 0.05 was considered statistically significant. R version 3.1.3 (R Development Core Team, Vienna, Austria) was used for all statistical analyses.

Ethical clearance was not required as the JTBS data do not include case identifiers, as according to the Ethical Guidelines for Epidemiological Research established by Ministry of Education, Culture, Sports, Science and Technology and Ministry of Health, Labour and Welfare of Japan.

## Results

### General trend

Between 2007 and 2014, of a cumulative total of 181 576 newly notified TB cases, 7832 were foreign-born (4.3%). The number of newly notified foreign-born TB patients has steadily increased from 842 in 2007 to 1101 in 2014. The proportion of foreign-born patients among the newly notified TB patients has also steadily increased (**Fig. 1a**) with the most prominent rise among 20–29 years olds (**Fig. 1b**). In 2014, 44.1% of TB cases among those aged 20–29 were foreign-born.

**Fig. 1a:**
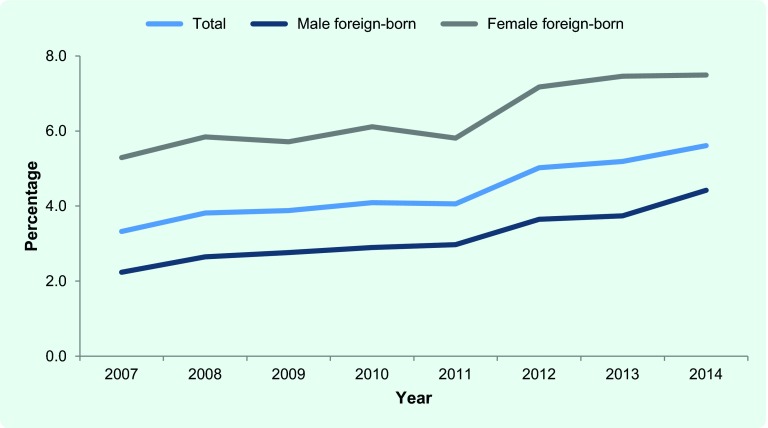
Foreign-born TB cases among newly notified TB patients, Japan, 2007–2014

**Fig. 1b:**
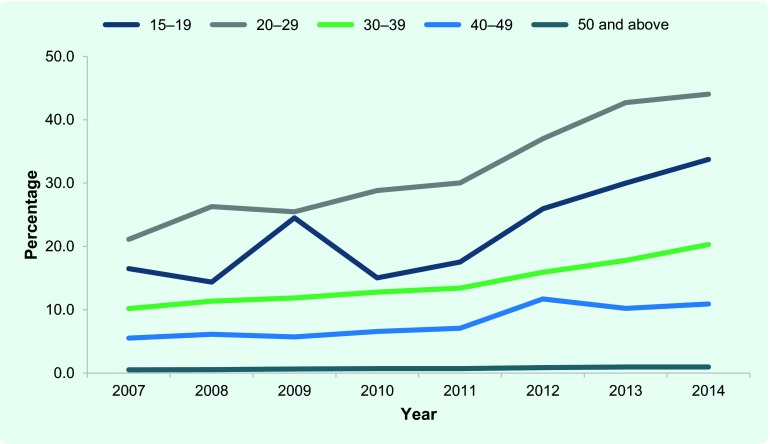
Foreign-born TB cases among newly notified TB patients by age group, Japan, 2007–2014

### Country of birth

Major countries of birth (or nationality, before 2012) among the foreign-born patients who entered Japan within the past five years and those who entered Japan more than five years ago are summarized in **Fig. 2a** and **2b**. The largest two groups have consistently been those from China and the Philippines. China occupied a greater share among the recently arrived TB patients (China, 32.7%; the Philippines, 15.2%; **Fig. 2a**), while those from the Philippines occupied a greater share among those who have entered Japan more than five years ago (China, 18.3%; the Philippines, 31.6%; **Fig. 2b**). The proportions of those from Nepal and Viet Nam have significantly increased among the recently arrived TB patients (Nepal; *P* < 0.01, χ^2^ = 45.9, Viet Nam; *P* < 0.01, χ^2^ = 42.6).

**Fig. 2a:**
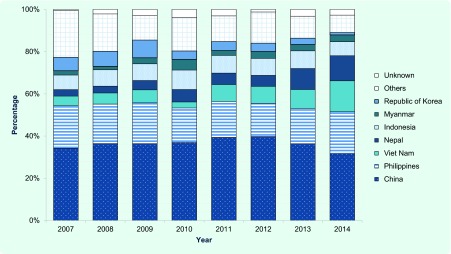
Countries of birth among foreign-born TB patients who entered Japan within five years, 2007–2014

**Fig. 2b:**
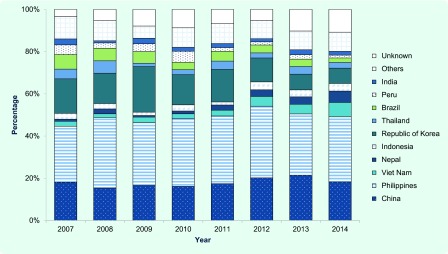
Countries of birth among foreign-born TB patients who entered Japan more than five years ago, 2007–2014

### Professional category

**Fig. 3:**
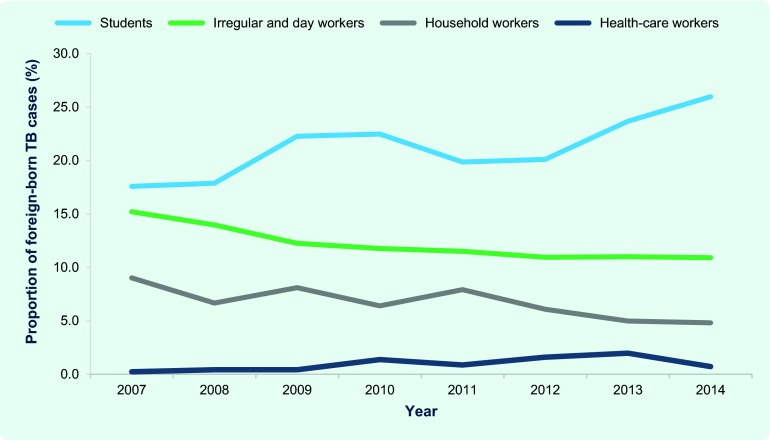
Proportion of selected professional categories among foreign-born TB patients, Japan, 2007–2014

Of the cumulative total of 7832 foreign-born patients between 2007 and 2014, 26.3% of them were regular workers and 21.4% were students (**Table 2**). The proportions of foreign-born TB patients among students and health-care workers have significantly increased (students, *P* < 0.01, χ^2^ = 21.7; health-care workers, *P* < 0.01, χ^2^ = 11.2), while those of irregular and day workers, and household workers have significantly decreased (irregular and day workers, *P* < 0.01, χ^2^ = 12.4; household workers; *P* < 0.01, χ^2^ = 17.0) (**Fig. 3**). Proportions of other professional categories remained constant.

**Table 2:** Professional categories of foreign-born TB patients, Japan, 2007–2014

Professional categories	*n*	%
Regular workers	2062	26.3
Students	1676	21.4
Irregular and day workers	947	12.1
Household workers	520	6.6
Service industry workers	466	5.9
Self-employed	146	1.9
Health-care workers	77	1.0
Infants and pre-schoolchildren	66	0.8
Teachers	30	0.4
Unemployed and others	1551	19.8
Unknown	291	3.7
Total	7832	100.0

### Mode of detection

Of the cumulative total of 7832 foreign-born TB patients, 67.2% were diagnosed while visiting medical institution with TB or other symptoms or during hospitalization for other diseases; whereas 22.3% were diagnosed through routine medical check-up (**Table 3**).

**Table 3:** Mode of TB case detection of Japan- and foreign-born TB patients, Japan, 2007–2014

Mode of detection	Japan-born		Foreign-born	
	*n*	%	*n*	%
Visit to medical institution/while being hospitalized for other illness	139 506	82.7	5264	67.2
Routine medical check-up	17 061	10.1	1747	22.3
Contact investigation	5086	3.0	333	4.3
Individual medical check-up	3657	2.2	185	2.4
Medical check-up during TB follow-up	629	0.4	26	0.3
Other mass medical check-up	522	0.3	147	1.9
Others	1159	0.7	62	0.8
Unknown	1021	0.6	68	0.9
Total	168 641	100.0	7832	100.0

**Fig. 4:**
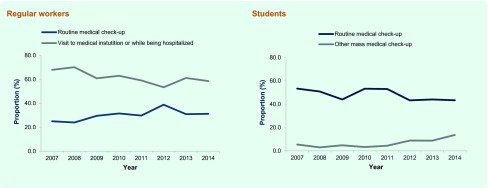
Mode of TB case detection among foreign-born TB patients by selected professional categories, Japan, 2007–2014

Stratified by students and regular workers, a significantly higher proportion of foreign-born students was diagnosed through routine or other mass medical check-ups (ad hoc medical check-up usually organized by PHCs) than the Japan-born students. On the other hand, a significantly lower proportion of foreign-born regular workers was diagnosed through routine medical check-up than their Japan-born counterparts (**Table 4**). However, as shown in **Fig. 4**, the proportion of foreign-born students diagnosed through routine medical check-up had significantly decreased throughout the study years (*P* = 0.01, χ^2^ = 6.1), while the proportion of those diagnosed through other mass medical check-ups had significantly increased (*P* < 0.01, χ^2^ = 23.9). The proportion of foreign-born regular workers diagnosed through routine medical check-up had significantly increased (*P* < 0.01, χ^2^ = 8.2) while the proportion of those diagnosed while visiting medical institution had significantly decreased (*P* < 0.01, χ^2^ = 12.8).

**Table 4:** Mode of TB case detection among Japan- and foreign-born TB patients by selected professional categories, Japan, 2007–2014

Mode of detection	Students			Regular workers		
	Japan-born (%)	Foreign-born (%)	*p-*value*	Japan-born (%)	Foreign-born (%)	*p-*value*
Individual medical check-up	3.8	2.4	< 0.05	2.7	1.7	< 0.05
Routine medical check-up	37.1	47.5	< 0.01	29.4	25.5	< 0.05
Contact investigation	11.5	3.9	< 0.01	5.7	5.1	0.16
Other mass medical check-up	1.0	7.0	< 0.05	0.3	0.3	0.98
Visit to medical institution/while being hospitalized for other illness	44.3	37.4	< 0.01	60.4	66.3	< 0.05
Medical check-up during TB follow-up	1.0	0.2	< 0.05	0.5	0.4	0.53
Others	0.4	0.6	0.44	0.4	0.3	0.55
Unknown	0.9	1.0	0.18	0.5	0.4	0.29
Total	100.0	100.0		100.0	100.0	

* By χ^2^ test.

### Treatment outcome

Of the cumulative total of 5353 foreign-born patients between 2007 and 2013, 53.4% had successfully completed treatment while 11.5% had transferred out. Compared with the Japan-born patients, after adjusting for age, the proportion of “success” and “treatment exceeding 12 months” was significantly lower, and “transferred-out” and “unknown” significantly higher among the foreign-born patients (**Table 5**).

**Table 5:** Treatment outcomes of Japan- and foreign-born TB patients, Japan, 2007–2013*

Treatment outcome	Japan-born		Foreign-born		Adjusted rate ratio (95%CI)
	*n*	%^?^	*n*	%^†^	
Success	63 334	62.6	3 091	53.4	0.85 (0.81–0.90)
Died	18 856	6.1	89	5.2	0.86 (0.70–1.06)
Treatment failed	815	0.7	30	0.7	1.05 (0.59–1.87)
Lost to follow-up	8 645	7.7	402	6.9	0.90 (0.77–1.06)
Transferred out	2 795	3.4	706	11.5	3.40 (2.88–4.01)
Treatment exceeding 12 months	11 545	8.5	349	7.2	0.84 (0.71–0.98)
Unknown	14 462	11.0	686	15.1	1.37 (1.17–1.60)
Total	120 452	100.0	5 353	100.0	

* The cohort data were only available until 2013.

^†^ Adjusted by age, using the population census of 2010. (*6*)

CI, confidence interval.

### Multidrug-resistant (MDR)-TB and HIV co-infection

**Fig. 5:**
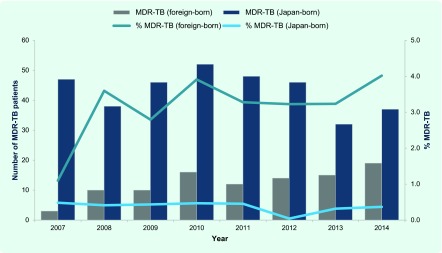
MDR-TB cases among Japan- and foreign-born TB patients, 2007–2014

The proportion of MDR-TB among the foreign-born TB patients was significantly higher than that among the Japan-born patients (3.2% versus 0.2%) over the years 2007–2014. The number of TB cases was also on the increase (**Fig. 5**). Of the cumulative total of 99 foreign-born MDR-TB patients, 44.4% (*n* = 44) were from China and 13.1% (*n* = 13) from the Philippines.

**Fig. 6:**
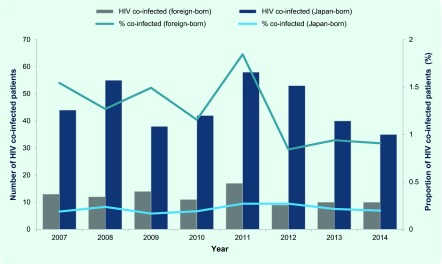
HIV co-infected cases among Japan- and foreign-born TB patients, Japan, 2007–2014

The proportion of HIV co-infected cases also had been significantly higher among the foreign-born patients than among the Japan-born TB patients (1.2% versus 0.2%) between 2007 and 2014; however no obvious increase was observed in the proportion both among the foreign- and Japan-born patients during the study period (**Fig. 6**). Of the cumulative total of 96 foreign-born HIV co-infected patients, 16.7% (*n* = 16) were from Thailand, 11.5% (*n* = 11) from Myanmar and 10.4% (*n* = 10) from Brazil.

## Discussion

Compared to many other low TB incidence countries, the current burden of foreign-born TB patients was relatively low in Japan. However, this proportion has been increasing steadily, especially among the younger age groups. This may partially be explained by the drastic increase in the number of foreign-born people entering Japan to study at Japanese language schools, many of whom are young and from TB high-burden countries. According to one study, the number of foreign-born people travelling to Japan to study at language schools increased from 25 622 in 2011 to 44 970 in 2014 – an increase of 37.8%. (*7*) In 2015, students from China consisted the largest group (36.4%), followed by Viet Nam (30.9%) and Nepal (12.4%). However, over the past decade, the proportion of students from China has steadily declined while those from Viet Nam and Nepal have increased eleven-fold and fivefold, respectively. (*8*) These recent trends among Japanese language school students were clearly reflected in the countries of birth and the professional categories of foreign-born TB patients in this study. An increase in the number of Japanese language school students also explains the rising proportion of students diagnosed through other mass medical check-ups. While routine medical check-ups at universities and vocational schools are mandatory under Japanese law, check-ups at Japanese language schools are optional and often entrust the planning and implementation of medical check-ups to local PHCs.

To our knowledge, although no study has yet examined the risk factors for TB of foreign-born students in Japan, several studies have indicated that foreign-born students are prone to poor socioeconomic status and physical and mental stress after arriving in Japan. For example, a study on the health and welfare of Japanese language school students reported that 66.5% of the respondents had experienced sickness or injury since their arrival in Japan, and 10.1% were not covered by any medical insurance. Furthermore, 73.4% of them had part-time jobs; 59.5% had answered that they were working for economic reasons. (*9*) Others have reported that foreign-born students living in Japan suffer from various psychological stress and depression. (*10*, *11*)

The number of registered foreign-born workers in Japan has also increased from 486 000 in 2008 to 787 627 in 2014. (*12*) However, as of 2014, we did not observe a clear increase in the proportion of regular workers among the foreign-born TB patients. This is partially due to the rise in the number of foreign-born workers on “conditional work permits” (i.e. students working part-time). When they are diagnosed with TB, however, they are registered as “students” under the JTBS. Foreign-born workers who work under the “technical internship permit” should also be noted. This work permit, introduced in 1993, and officially designed to support mainly those from developing countries to acquire skills and knowledge of Japanese advanced technology, has been criticized from various domestic and international communities as a means for Japanese companies to secure a cheap foreign labour force. (*13*, *14*) It has also been reported that those on “technical internship permits” often live in poor social and economic conditions and with limited access to social and health care services in Japan. (*15*) In 2015, 41.9% of such workers came from Viet Nam, 29.6% from China and 11.8% from the Philippines. (*16*) The JTBS does not differentiate those workers on “technical internship permit” from other “regular” workers, and we cannot quantify the burden of TB among workers under this permit. However, considering the high TB burden in their home countries, and the socioeconomic vulnerability which they face in Japan, those on “technical internship permits” should be recognized as having a higher TB risk than other foreign-born people in Japan.

Treatment costs for most TB patients who require hospitalization, including those foreign-born, are subsidized by the Japanese government. Outpatients are requested to pay only 5% of their treatment cost and it is usually covered by their health insurance. The main barrier to TB treatment success is the high proportion of those who transfer out. Among the foreign-born patients, transferred-out cases mostly refer to those who have returned to their home country while they are still on treatment. Unlike “lost to follow-up” cases, whereby patients terminate treatment in Japan without informing the local PHC, “transferred-out” indicates that at least an effort was made to organize the transfer of patient from a medical institution in Japan to a relevant counterpart in the patients’ home country. However, as of today, no systematic arrangement exists to enable local PHCs in Japan to confirm treatment results of foreign-born patients who have transferred out of the country.

A significantly higher proportion of MDR-TB among the foreign-born patients was expected considering the high burden of MDR-TB in the home countries of those patients. On the other hand, the proportion of HIV co-infected among the foreign-born TB patients in Japan was low, reflecting the relatively low prevalence of HIV in Asia.

The limitations of our study reflect those inherent in the JTBS. Despite TB being a notifiable disease and the various mechanisms to ensure quality control of TB surveillance data, anecdotal evidence suggests underreporting as well as inaccurate or incomplete data entry in the system. For example, Uchimura has estimated the underreporting rate of the JTBS was approximately 5%. (*16*) Improvements to the system in the next few years may hopefully minimize these errors.

## Conclusion

A detailed analysis of surveillance data revealed that distinct subgroups within foreign-born populations in Japan, namely students and certain types of workers, were at a higher risk of developing TB. Measures to ensure early access to diagnosis and treatment, regardless of whether they choose to return to their home country, should be adapted to such populations for TB control and prevention.
